# A Dnmt3a-Mediated Epigenetic Mechanism for Chronic-Pain-Induced Behaviors of Negative Emotion in Mice

**DOI:** 10.3390/cells15141304

**Published:** 2026-07-21

**Authors:** Jianlong Ge, Youqing Cai, Zhizhong Z. Pan

**Affiliations:** Departments of Anesthesiology and Pain Medicine, The University of Texas M. D. Anderson Cancer Center, Houston, TX 77030, USA; jge@mdanderson.org (J.G.); youcai@mdanderson.org (Y.C.)

**Keywords:** Dnmt3a, emotion, glutamate receptors, adeno-associated virus, electrophysiology

## Abstract

The comorbidity of chronic pain and associated negative emotion significantly worsens pain experience and pain chronicity, presenting a severe clinical problem. Despite extensive pre-clinical and clinical studies, the molecular mechanisms underlying the pain–negative-emotion comorbidity are still poorly understood. In this study, we explored an epigenetic mechanism for chronic-pain-induced negative emotion, focusing on the DNA methyltransferase 3a (Dnmt3a) in mice with chronic neuropathic pain. In the parabrachial nucleus (PBN), a brainstem gateway to transmitting pain signals from the peripheral to the brain, we found that chronic pain lasting one month induced anxiety- and depression-like behaviors and downregulated Dnmt3a protein. Overexpression of PBN Dnmt3a rescued the chronic pain mice from developing these behaviors of negative emotion, while knockdown of PBN Dnmt3a was sufficient to induce anxiety-like behavior in naive mice. In contrast, chronic pain increased expression of GluA1-pSer831 subunits of glutamate AMPA receptors in PBN. Dnmt3a knockdown increased both the expression of PBN GluA1-pSer831 and synaptic function of AMPA receptors in PBN neurons. Overexpression of PBN GluA1 induced anxiety-like behavior in naïve mice, whereas knockdown of PBN GluA1 produced an anxiolytic effect and prevented chronic-pain-induced anxiety-like behavior. These results suggest Dnmt3a as a key epigenetic modulator linking chronic pain to negative emotion behaviors through PBN, and PBN GluA1 as an important regulation target of Dnmt3a in the process.

## 1. Introduction

It has been reported that about ten percent of the general population suffer from chronic neuropathic pain, which represents a considerable clinical and economic burden at the family and society level [1,2,3]. Patients with chronic neuropathic pain not only suffer from many painful symptoms but also often experience mood disorders such as anxiety and depression [4,5,6,7]. Negative mood state can increase pain sensitivity and chronicity, and vice versa, creating the comorbid disease of chronic pain that severely impairs patients’ quality of life [1,8]. Due to limited availability and efficiency of non-opioid strategies, many patients with chronic pain and comorbid mood disorders are maintained on long-term opioid regimens, which increases the risk of opioid misuse, dependence, and overdose [9,10,11]. Thus, identifying a new molecular target in the mechanisms for chronic- pain–negative-emotion comorbidity is important not only for improving the relief of pain and mood symptoms, but also for strengthening the public health effort to reduce opioid misuse and associated morbidity and mortality.

Among several ascending pain pathways are two that have been well characterized, as follows: the spinothalamic pathway transmitting primarily nociceptive pain signal from spinal cord to thalamus, and the spinoparabrachial pathway conveying mainly the emotional component of pain from spinal cord to the parabrachial nucleus (PBN) and then PBN’s projection targets [12]. However, recent studies suggest that both pathways can contribute to encoding of nociceptive signals and affective-emotional information with thalamic neurons carrying signals of affective pain and the spinoparabrachial pathway relaying nociceptive signals [13,14]. Previous studies with extensive anatomical and functional evidence have well established the roles of PBN in relaying pain signals from spinal cord to forebrain regions including the central nucleus of the amygdala (CeA), periaqueductal grey, and several hypothalamic nuclei [15,16]. Particularly, it has been shown that calcitonin gene-related peptide (CGRP)-expressing neurons in PBN contribute to an affective threat memory from pain in mice [17]; and activation of the projection from PBN to CeA induces anxiety and depression behaviors in rats [18]. Thus, PBN as a central hub for relay of affective pain signals is well documented [19]. While many studies have illustrated the cellular and circuit functions of PBN neurons in relaying signals of nociceptive and affective pain, what remain unclear are intracellular signaling mechanisms that contribute to the comorbidity of chronic pain and aversive emotion behaviors.

Recent research has revealed that epigenetic regulation of gene expression is a novel and key mechanism for the pathogenesis of many chronic neurological diseases including chronic pain [20,21,22,23]. Of the two major forms of chromatin modifications–histone acetylation and DNA methylation—in epigenetic regulation, DNA methylation for gene repression is more persistent, causing long-lasting changes in the landscape of gene expression [24,25]. Thus, DNA methylation is likely more important for the development of chronic pain with long-term changes in nociceptive and emotion behaviors. DNA methylation is catalyzed by three major DNA methyltransferases (Dnmts), as follows: Dnmt1 acts to maintain established DNA methylation patterns during DNA replication, and Dnmt3a and Dnmt3b mediate de novo DNA methylation [24,25,26]. In recent years, substantial evidence has emerged for important roles of DNA methylation and chromatin modifications in the mechanisms of chronic pain on various animal models [27,28]. As for the specific regulators for DNA methylation, recent studies have shown the role of Dnmt3a in the maintenance and sensitization of acute sensory pain in peripheral sensory neurons [29,30,31,32]. However, it remains unclear how the Dnmt3a-dependent DNA methylation mechanisms are involved in the PBN functions conveying the affective dimension of chronic pain. In this study, we explored the role of Dnmt3a and its potential regulation target in the development of chronic-pain-induced behaviors of negative emotion in a mouse model of chronic neuropathic pain.

## 2. Methods

### 2.1. Animals

Adult C57BL/6J mice and *Dnmt3a*^fl/fl^ mice of both sexes weighing 15–25 g were used in this study. Initial studies and data analysis did not reveal potential sex differences in the parameters measured, therefore data of both sexes were pooled. Breeding pairs of *Dnmt3a*^fl/fl^ mice were kindly provided by Dr. Yuanxiang Tao at The State University of New Jersey, NJ and bred in our animal facility. Animals were randomly assigned to control groups and treatment groups. Group size was estimated by a power analysis and adjusted to reach statistical significance with sufficient power and a minimum number of animals used. The experimenter was blind to animal groups of control and treatments. An animal was excluded from the study only when a health issue developed, as determined by a veterinarian, that would confound the results during the course of an experiment. All procedures involving the use of animals are conducted in accordance with the guidelines and approved by our institutional Animal Care and Use Committee (approved protocol # 00000834-RN04). Mice were housed in a 12 h light/dark cycle with food and water available ad libitum. All behavioral experiments and tests were performed between 8:00 a.m. and 18:00 p.m.

The behavioral assays for pain and emotion were performed sequentially on the same groups of animals for each experiment. The same emotion assay for anxiety- and depression-like behaviors was conducted only once on each animal.

### 2.2. The Spared Nerve Injury Model of Chronic Neuropathic Pain

The spared nerve injury (SNI) was performed to induce the mouse model of chronic neuropathic pain [33]. Mice were anesthetized under isoflurane anesthesia. The skin was shaved on the lateral surface of the left thigh with a razor blade, followed by a small skin incision about 1 cm at the mid-thigh level. Blunt dissection was made through the biceps femoris muscle to expose the sciatic nerve and its three branches. The common peroneal and tibial nerves were ligated with 8-0 nylon suture and the sural nerve was left intact. The ligated nerves were then transected distally by removing a 2–4 mm piece of each distal nerve stump to avoid nerve regeneration. In sham-operated control mice, the sciatic nerve was only exposed without ligation and transection. After the surgery, the mice were monitored closely until full recovery before being returned to their home cages.

### 2.3. Test for Thresholds of Mechanical Pain

A mouse was placed in a plastic box with mesh floor and allowed to acclimate for 10 min. A series of calibrated von Frey filaments were applied perpendicularly to the plantar surface of a hind paw with sufficient force to bend the filament. A brisk movement of the hind paw (withdrawal or flinching) was considered as a positive response. A 50% threshold was computed by an adaptive response sequence with the “up-down” calculating method [34]. The paw-withdrawal response was measured twice with a 5 min interval, and the average of the two measurements were used as data points.

### 2.4. Open Field Test

The open field test (OFT) is a classical test to measure anxiety-like behaviors and general locomotor activity in rodents [35,36]. In an illuminated arena (72 × 72 × 30 cm) divided by a central zone and an outer zone (area ratio 1:3), a mouse was placed in the center of the arena and its locomotor activity in the two zones was recorded for 15 min and analyzed by an automated video-tracking system (EthoVision XT, Noldus Information Technology, Wageningen, The Netherlands). Reduced time spent and distance traveled in the unprotected central zone were regarded as indices of anxiety-like behavior. Total distance traveled in the entire arena was also recorded and used as a measure of locomotor activity.

### 2.5. Elevated Plus Maze Test

The elevated plus maze test (EPMT) is another widely used measure for anxiety in rodents and the first-choice test for screening anxiolytic drugs [36,37,38]. The EPMT apparatus consisted of two opposite open arms and two closed arms (31 × 6.5 × 15 cm) elevated 38 cm above the floor. In an EPMT, a mouse was placed in the junction of the four arms facing an open arm and was allowed 5 min of free exploration. Time spent in open arms (expressed as % of time spent in the open arms vs. the time spent in all arms) and total distance traveled during the 5 min test period were recorded and analyzed by the video-tracking system. A decrease in the time spent in the open arms was considered as an index of anxiety-like behavior.

### 2.6. Forced Swim Test

The forced swim test (FST) is a main behavioral test for depression-like behaviors in rodents and for screening antidepressant drugs [35]. A mouse was placed in a clear plastic cylinder filled with water (20–30 cm deep, 25 ± 1 °C) for 6 min and was recorded with the video-tracking system. Time the animal spent immobile during the test was considered as a measure of depression-like behaviors. Immobility was defined as cessation of all active swimming and escaping activities.

### 2.7. Western Blots

After behavioral assays, mice were deeply anesthetized by isoflurane and then decapitated. The brain was cut in a vibratome in cold artificial cerebrospinal fluid to obtain brain slices (0.6 mm thick). Both sides of targeted PBN were isolated and the tissues were homogenized with RIPA lysis buffer and placed on ice for 1 h, followed by centrifuging at 12,000 rpm × 4 °C for 20 min. The supernatant was collected and the Lorry assay was performed to determine protein concentration. Samples containing equal protein amount (10–50 µg) were loaded and separated on an 8% SDSPAGE gel. Five percent non-fat milk was used to block non-specific binding sites in polyvinylidene difluoride membranes (PVDF) with transferred protein for 1 h. Then the blots were incubated with a primary antibody against Dnmt3a (D23G1, Cell Signaling, Danvers, MA, USA, 1:1000, RRID: AB_2277449), Dnmt3b (PA5-32218, Thermo Fisher Scientific, Waltham, MA, USA, 1:1000, RRID: AB_2549691), Dnmt1 (D63A6, Cell Signaling, 1:1000, RRID: AB_2799659), GluA1-pSer831 (04-823, Millipore, Darmstadt, Germany, 1:1000, RRID: AB_1977218), GluA1 (AGC-004, Alomone labs, Jerusalem, Israel, 1:1000, RRID: AB_2039878), GluA2 (ab20673, Abcam, Cambridge, UK, 1:1000, RRID: AB_2232655), or β-actin (sc-81178, Santa Cruz Biotechnology, Dallas, TX, USA, 1:1000, RRID: AB_2223230) overnight at 4 °C. The horseradish peroxidase-conjugated secondary antibody (1:10,000; Jackson Immuno Research, West Grove, PA, USA) was then applied for 1 h. The blots were developed with ECL plus reagent (GE Healthcare, Chicago, IL, USA). Image J software was used to determine the densitometric quantification of immunoreactive bands. All levels of targeted proteins were normalized to β-actin.

### 2.8. Quantitative Real-Time-PCR

Total RNA was extracted from the PBN of SNI and sham mice by the PureLink™ Total RNA Purification System (Invitrogen, Carlsbad, CA, USA) with on-column DNase I digestion performed according to the manufacturer’s instructions. For each sample, 2 µg of total RNA was reverse-transcribed at 37 °C for 60 min with random primers and M-MLV reverse transcriptase. cDNA was synthesized with the SuperScript™ III First-Strand Synthesis Kit (Invitrogen, Carlsbad, CA, USA). Quantitative PCR was performed with the ABI 7500 Real-Time PCR System and the Power SYBR™ Green PCR Kit (Life Technologies, Carlsbad, CA, USA). All samples were run in duplicate with an annealing temperature of 60 °C, and each experiment was repeated at least once. The primer sequences used were as follows: mouse Dnmt3a (NM_007872), forward 5′-GCAGAAACATCGAGGACATTTG-3′ (1689–1701) and reverse 5′-CTGGTAAGCACACTCCAAGAA-3′ (1810–1789, efficiency 99.2%); mouse GAPDH (GU214026), forward 5′-AGGTCGGTGTGAACGGAT-3′ (79–96) and reverse 5′-TGTAGACCATGTAGTTGAGGTCA-3′ (201–179, efficiency 102.3%). Relative Dnmt3a mRNA expression levels were calculated by the 2^−ΔΔCt^ method and normalized to the housekeeping gene GAPDH. The mean mRNA level in sham mice was set to 1. PCR specificity was confirmed by melting curve analysis and agarose gel electrophoresis.

### 2.9. Adeno-Associated Viral Vectors and Viral Injection into PBN

Adeno-associated virus (AAV)-CaMKIIα-GFP and AAV-CaMKIIα-Cre vectors were obtained from the Vector Core Facility at The University of North Carolina at Chapel Hill. AAV-CMV-Dnmt3a (Cat# AAV-257356) and AAV-CMV-GFP (Cat# 7006) were obtained from Vector Biolabs. For construction of AAV-CMV-GluA1 to overexpress GluA1, PCR product of coding area of GluA1-flip version was amplified with the following primer pair: 5′-CCGAATTCTATGCCGTACATCTTTGCC-3′ and 5′ CCGTCGACTTACAATCCTGTGGCTCCCAAGG-3′, purified, and inserted into pAAV-CMV backbone vector. It was further validated by expression of GluA1 protein in CHO cells. For construction of AAV-GluA1-shRNA vector to knockdown GluA1, the shRNA sequence 5′-GGAATCCGAAAGATTGGTTAC-3′ was selected (designed with BLOCK-iT RNAi Designer, Invitrogen) and cloned into pAAVsc-si-EGFP shRNA expression vector with a loop sequence of TTCAAGAGA. The silence efficiency was evaluated by co-transfection of each shRNA expression vector with AAV-GluA1 vector into CHO cells. Two weeks after the sham and SNI surgery, the viral vector was bilaterally injected (0.5 μL each side) into PBN (anteroposterior, −5.0 mm from the bregma; lateral, ±1.1 mm; ventral, −3.5 mm from dura) in mice under anesthesia in a stereotaxic instrument. Behavioral experiments were performed 2–3 weeks after the vector injection. After the experiments, brain slices (150 µm thick) were obtained and the injection site was anatomically verified by the location of GFP expression of the injected viral vector under a fluorescent microscope.

### 2.10. Whole Cell Recording

The detailed methods for whole-cell recordings and synaptic analyses have been described in our previous study [39]. Briefly, PBN-containing brain slices (250 µm thick) were obtained for whole-cell recordings on PBN neurons. For miniature inhibitory postsynaptic current (mIPSC), the holding potential was −70 mV, resulting in downward currents with a KCL-filled recording electrode. Excitatory postsynaptic currents (EPSCs) were evoked electronically. The AMPA EPSC was recorded in D-AP5 (50 μM) and the NMDA EPSC was determined by subtracting the AMPA EPSC from a total EPSC to obtain the AMPA EPSC/NMDA EPSC ratio. All evoked and miniature synaptic currents were confirmed by selective pharmacological antagonists of the respective receptors.

### 2.11. Data and Statistical Analysis

Numerical data were analyzed and tested statistically with statistical methods appropriate for different experimental settings and data sampling. The standard non-paired, two-tailed Student’s *t* test was used for simple comparison between the two groups to determine statistical difference. One-way ANOVA was utilized for statistical comparison among three or more groups. Two-way ANOVA was employed to determine statistical difference in data from experiments with repeated measures on the same subjects. The Bonferroni test was used for post hoc analyses in two-way ANOVA. The Prism 10 software was used in all the tests. Statistical significance was confirmed by a *p* value < 0.05. Data were expressed as means ± S.E.M.

## 3. Results

### 3.1. Chronic Pain Induces Behaviors of Negative Emotion with Decreased Dnmt3a in PBN

**Figure 1:**
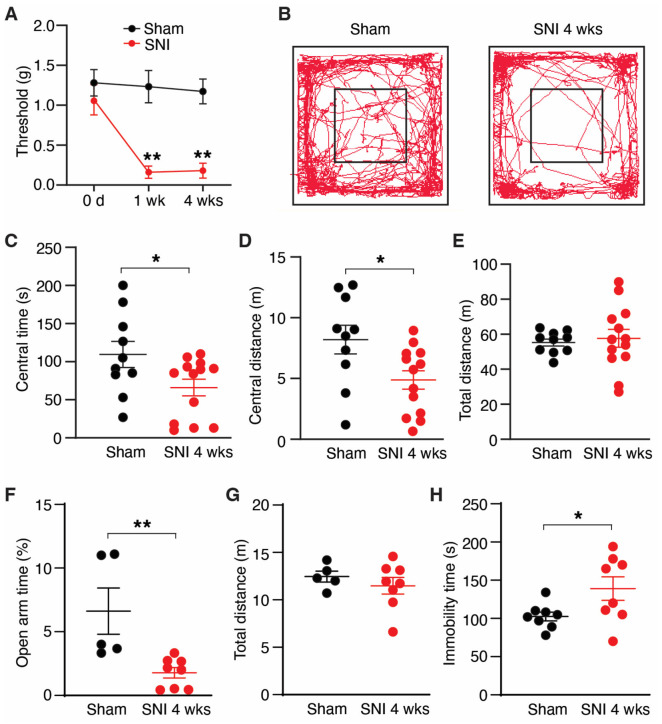
Chronic pain induces anxiety- and depression-like behaviors in the parabrachial nucleus (PBN). (**A**) Thresholds of mechanical pain in mice 1 and 4 weeks after spared nerve injury (SNI) or sham surgery (two-way ANOVA with the Bonferroni post hoc test. (**B**) Locomotion traces of a mouse from the sham group and from the SNI group 4 weeks after the surgery in the Open Field Test (OFT). The inner squares mark the central zone. (**C**–**E**) Group data of time spent (**C**) and distance traveled (**D**) in central zone and total distance traveled in the entire arena (**E**) in mice from sham group and SNI group 4 weeks after the surgery. (**F**,**G**) Group data of time spent in open arms (as % of the time spent in all arms, (**F**) and total distance traveled (**G**) in the elevated plus maze test (EPMT) in sham-operated and SNI-operated mice 4 weeks after the surgery. (**H**) Immobility time in the forced swim test (FST) in the sham and SNI groups of mice. * *p* < 0.05, ** *p* < 0.01.

After the SNI surgery, the mice displayed long-lasting, hypersensitized nociceptive response (mechanical allodynia) with significantly decreased pain thresholds when compared with the sham-operated mice [Day (changes over 4 weeks), F_(2, 12)_ = 6.52, *p* = 0.01, SNI (changes in SNI group compared to sham group), F_(1, 6)_ = 31.45, *n* = 6, *p* < 0.01, two-way ANOVA with the Bonferroni post hoc test, Figure 1A]. The hypersensitivity lasted at least one month with no apparent sign of recovery, confirming a model of chronic neuropathic pain. We then examined whether the chronic pain would induce a change in emotional behaviors such as anxiety and depression. Using the open field test (OFT), we found that the mice with chronic pain displayed significant anxiety-like behavior with significantly decreased travel time and travel distance in the central zone (central time and central distance, respectively), as measured 4 weeks after the SNI or sham surgery (Central time: sham, 109.5 ± 17.0 s, *n* = 10, SNI, 66.0 ± 10.9 s, *n* = 13, *p* = 0.03; central distance: sham, 8.20 ± 1.18 m, *n* = 10, SNI, 4.88 ± 0.76 m, *n* = 13, *p* = 0.02, unpaired *t* test, Figure 1B–D). There was no significant difference in total distance traveled in the entire arena between the sham and SNI groups (Total distance: sham, 55.28 ± 1.96 m, *n* = 10, SNI, 57.64 ± 5.13 m, *n* = 13, *p* = 0.7, Figure 1E), suggesting that the chronic neuropathic pain did not affect the locomotor activity of the mice. This chronic pain-induced anxiety behavior was further confirmed by the elevated plus maze test (EPMT), as the mice with chronic pain spent significantly less time in the unprotected open arm (open arm time) when compared with the sham mice with no significant difference in total distance travelled between the two mouse groups (Open arm time: sham, 6.62 ± 1.81%, *n* = 5, SNI, 1.78 ± 0.41%, *n* = 8, *p* = 0.007; Total distance: sham, 12.45 ± 0.58 m, *n* = 5, SNI, 11.48 ± 0.87 m, *n* = 8, *p* = 0.43, unpaired *t* test, Figure 1F,G). In addition, we tested depression-like behavior with the forced swim test (FST). Our results showed that chronic pain mice displayed significantly more immobility time than the sham mice (sham, 102.5 ± 5.8 s, *n* = 8, SNI, 139.1 ± 15.3 s, *n* = 8, *p* = 0.04, unpaired *t* test, Figure 1H). Together, these results indicate that chronic pain may induce a state of negative emotion with anxiety- and depression-like behaviors in mice.

**Figure 2:**
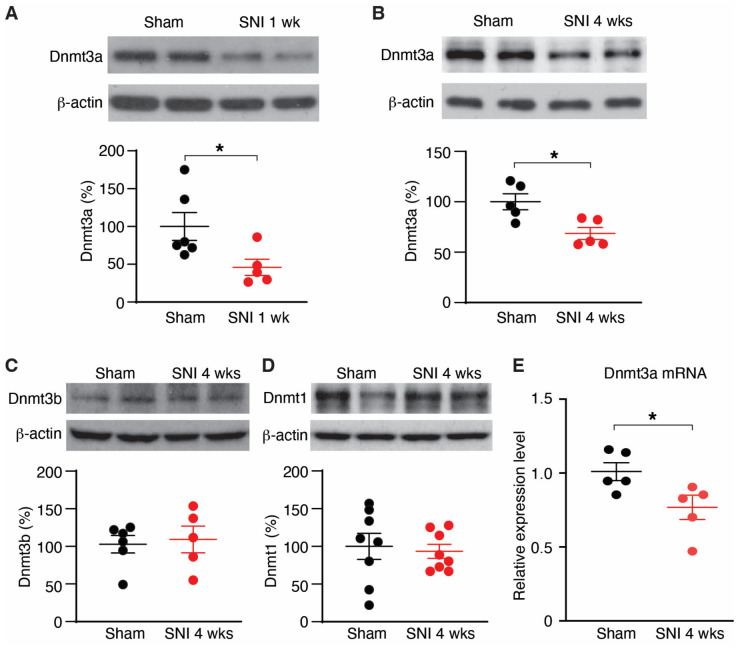
Chronic pain downregulates Dnmt3a protein and mRNA in PBN. (**A**–**D**) Western blots and group data of the protein levels of DNA methyltransferase 3a (Dnmt3a, (**A**,**B**)), Dnmt3b (**C**) and Dnmt1 (**D**), normalized to that of b-actin in PBN from sham mice and SNI mice 1 week (**A**) and 4 weeks (**B**–**D**) after the surgery. (**E**) Normalized expression level of Dnmt3a mRNA in the PBN from sham mice and SNI mice 4 weeks after the surgery. * *p* < 0.05.

Next, we determined whether an epigenetic mechanism was involved in the chronic- pain-induced behaviors of negative emotion, focusing on Dnmt3a, Dnmt3b, and Dnmt1 for DNA methylation in PBN. We found that the expression level of Dnmt3a in PBN significantly decreased 1 week and 4 weeks after SNI surgery when compared with the sham group (1 week: sham, 100.0 ± 18.4%, *n* = 6, SNI, 45.9 ± 10.7%, *n* = 5, *p* = 0.03; 4 week: sham, 100.0 ± 7.9%, *n* = 5, SNI, 68.5 ± 5.9%, *n* = 5, *p* = 0.01, unpaired *t* test, Figure 2A,B). In contrast, the protein levels of Dnmt3b and Dnmt1 in PBN were unchanged by the SNI 4 weeks after the surgery (Dnmt3b: sham, 100.0% ± 11.3%, *n* = 6, SNI, 106.5 ± 17.2%, *n* = 5, *p* = 0.75; Dnmt1: sham, 100.0 ± 17.2%, *n* = 8, SNI, 93.4 ± 9.3%, *n* = 8, *p* = 0.74, unpaired *t* test, Figure 2C,D). We also examined Dnmt3a mRNA using quantitative real-time PCR. Our data showed that the relative level of Dnmt3a mRNA in PBN was significantly decreased in SNI mice 4 weeks after the surgery (Sham, 1.01 ± 0.13, *n* = 5, SNI, 0.77 ± 0.18, *n* = 5, *p* < 0.05, unpaired *t* test, Figure 2E), consistent with the Western blot results above. Thus, it appears that, among the major Dnmts, Dnmt3a in PBN may be the primary epigenetic regulator involved in the anxiety- and depression-like behaviors in mice with chronic pain.

### 3.2. Overexpression of PBN Dnmt3a Relives Chronic-Pain-Induced Behaviors of Negative Emotion

**Figure 3:**
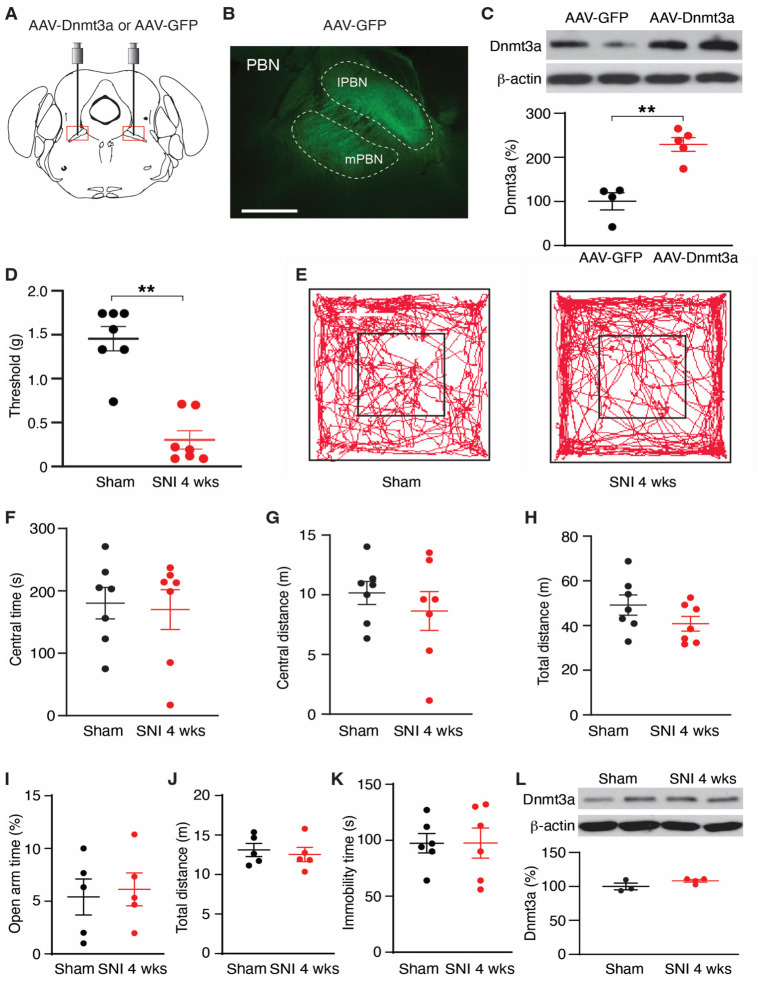
Overexpression of PBN Dnmt3a rescues mice from chronic-pain-induced anxiety- and depression-like behaviors. (**A**) A diagram illustrating bilateral microinjection of the control vector AAV-CMV-GFP or the AAV-CMV-Dnmt3a vector into PBN (indicated by red squares) to overexpress local Dnmt3a in mice. (**B**) A representative image of a PBN injection site and GFP-labeled PBN from an AAV-CMV-GFP-injected mouse. Scale bar is 500 µm. (**C**) Western blots and normalized group data of Dnmt3a protein levels in PBN in mice 2 weeks after PBN injection of AAV-GFP or AAV-Dnmt3a. (**D**) Pain thresholds in sham and SNI mice (4 weeks after the surgery) with overexpression of PBN Dnmt3a 2 weeks after PBN injection of AAV-Dnmt3a in both groups. (**E**–**H**) Locomotion traces of a mouse (**E**), and group data of time spent (**F**) and distance traveled (**G**) in central zone, and total distance traveled in the entire arena (**H**) in OFT from sham group and SNI group (4 weeks after the surgery) with PBN Dnmt3a overexpression (2 weeks after PBN injection of AAV-Dnmt3a in both groups). (**I**–**K**) Group data of open arm time (**I**) and total distance traveled (**J**) in EPMT, and immobility time in FST (**K**) from sham and SNI groups after overexpression of PBN Dnmt3a. (**L**) Western blots and group data of normalized protein levels of Dnmt3a in PBN in mice from sham group and SNI group (4 weeks after the surgery) with PBN Dnmt3a overexpression in both groups (2 weeks after PBN injection of AAV-Dnmt3a). lPBN, lateral PBN. mPBN, medial PBN. ** *p* < 0.01.

To verify this role of PBN Dnmt3a, we used a viral vector to overexpress Dnmt3a locally in PBN and examined its effects on nociceptive response and behaviors of negative emotion. AAV-CMV-DNMT3a or the control vector AAV-CMV-GFP was bilaterally microinjected into PBN of mice (Figure 3A). Figure 3B shows a typical injection site for PBN with GFP signals expressed exclusively within PBN two weeks after injection of AAV-CMV-GFP. Two weeks after PBN injection of AAV-CMV-Dnmt3a or AAV-CMV-GFP in naïve mice, the protein level of Dnmt3a in PBN normalized to that of b-actin was significantly increased in the AAV-CMV-Dnmt3a-injected group when compared with the control group (AAV-GFP, 100.0 ± 19.7%, *n* = 4, AAV-Dnmt3a, 229.3 ± 15.6%, *n* = 5, *p* < 0.01, unpaired *t* test, Figure 3C). This result confirms the successful overexpression of Dnmt3a in PBN. Then we determined the behavioral effects of this Dnmt3a overexpression on nociceptive response and behaviors of negative emotion. Two weeks after bilateral injection of AAV-CMV-Dnmt3a into the PBN of SNI and sham mice, we found that the SNI-operated mice still displayed similar nociceptive hypersensitivity with significantly decreased mechanical pain thresholds 4 weeks after the SNI surgery (Sham, 1.45 ± 0.13 g, *n* = 7, SNI, 0.30 ± 0.10 g, *n* = 7, *p* < 0.01, unpaired *t* test, Figure 3D). However, in strong contrast, we found that the chronic SNI was no longer able to induce anxiety-like behavior after overexpression of PBN Dnmt3a in OFT (Central time: sham, 180.4 ± 25.2 s, *n* = 7, SNI, 170.0 ± 31.9 s, *n* = 7, *p* = 0.8; central distance: sham, 10.16 ± 0.96 m, *n* = 7, SNI, 8.64 ± 1.63 m, *n* = 7, *p* = 0.4, unpaired *t* test, Figure 3E–G) with no change in the total distance traveled (sham, 49.18 ± 4.52 m, *n* = 7, SNI, 40.80 ± 3.29 m, *n* = 7, *p* = 0.15, unpaired *t* test, Figure 3H). Similar results were obtained in EPMT with no significant anxiety-like behavior after the Dnmt3a overexpression in the SNI group (Open arm time: sham, 5.40 ± 1.70%, *n* = 5, SNI, 6.13 ± 1.56%, *n* = 5, *p* = 0.76, unpaired *t* test, Figure 3I). The total distance traveled in EPMT remained unchanged between the two mouse groups (sham, 13.1 ± 0.82 m, *n* = 5, SNI, 12.5 ± 0.90 m, *n* = 5, *p* = 0.65, unpaired *t* test, Figure 3J). Chronic SNI also failed to induce depression-like behavior after overexpression of PBN Dnmt3a as measured in FST (Immobility time: sham, 97.3 ± 8.7 s, *n* = 6, SNI, 97.5 ± 13.4 s, *n* = 6, *p* = 0.99, unpaired *t* test, Figure 3K). Bilateral injection of the control GFP vector did not affect either nociceptive response or emotion behaviors, as shown in our previous studies [18,40,41]. These behavioral results were further supported by our Western blot experiments, which showed that overexpression of PBN Dnmt3a overcame the effect of chronic SNI in decreasing Dnmt3a in PBN, with no difference in the Dnmt3a levels between the pain and control groups of mice (sham, 100.0 ± 4.9%, *n* = 3, SNI, 108.2 ± 2.0%, *n* = 4, *p* = 0.14, unpaired *t* test, Figure 3L). These results support the notion that chronic pain decreases the expression of Dnmt3a in PBN, which likely induces a behavioral state of negative emotion, as overexpression of PBN Dnmt3a prevents the mice from developing these behaviors of negative emotion, but not from chronic SNI-induced nociceptive hypersensitivity.

### 3.3. Knockdown of PBN Dnmt3a Induces Anxiety-like Behavior

**Figure 4:**
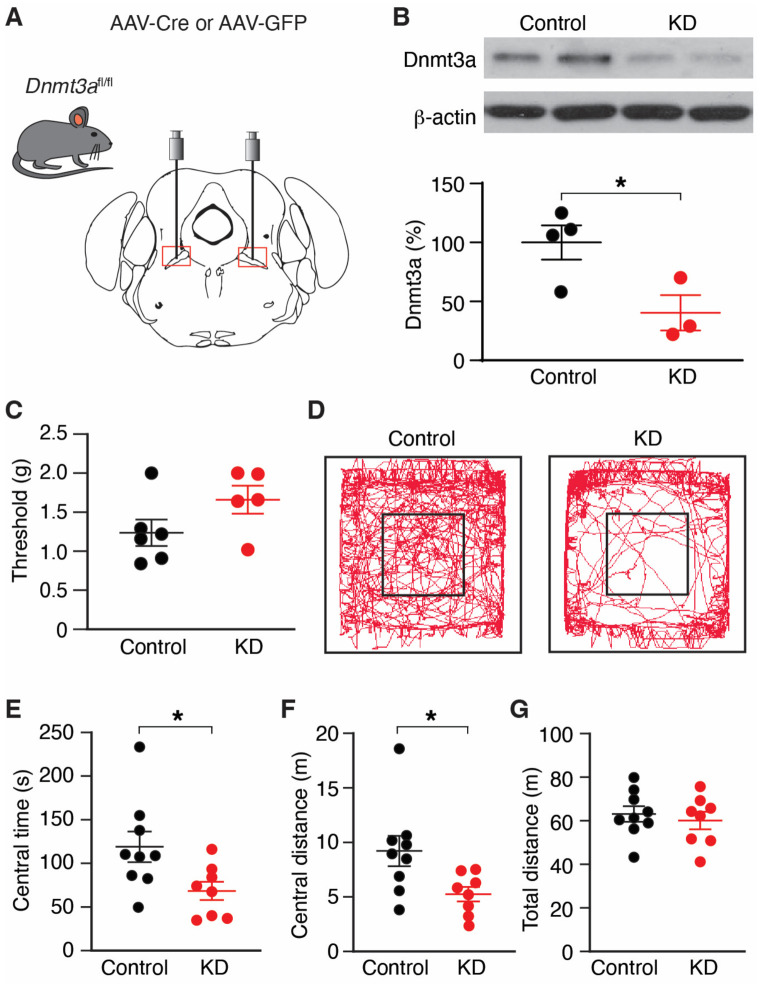
Knockdown of PBN Dnmt3a induces anxiety-like behavior. (**A**) A diagram illustrating bilateral injection of the viral vector AAV-CaMKII-Cre or the control vector AAV-CaMKII-GFP into the PBN of *Dnmt3a*^fl/fl^ mice. (**B**) Western blots and group data of normalized Dnmt3a protein levels in the *Dnmt3a*^fl/fl^ mice of control group with PBN injection of AAV-CaMKII-GFP and of knockdown (KD) group with PBN injection of AAV-CaMKII-Cre 3 weeks after the PBN injections. (**C**) Pain thresholds in the *Dnmt3a*^fl/fl^ mice of AAV-GFP-injected control group and AAV-Cre-injected knockdown group 3 weeks after the PBN injections. (**D**–**G**) Locomotion traces of a mouse (**D**), and group data of time spent (**E**) and distance traveled (**F**) in central zone, and total distance traveled in the entire arena (**G**) in OFT in the *Dnmt3a*^fl/fl^ mice of AAV-GFP-injected control group and AAV-Cre-injected knockdown group 3 weeks after the PBN injections. * *p* < 0.05.

To further verify the causal role of PBN Dnmt3a in chronic-pain-induced anxiety, we used the Cre-dependent *Dnmt3a*^fl/f^ mice to knockdown local Dnmt3a in PBN and determined its behavioral effects on nociceptive response and anxiety behavior. Three weeks after bilateral injection of AAV-CaMKII-Cre virus into the PBN of *Dnmt3a*^fl/f^ mice (Figure 4A), we found that the protein level of PBN Dnmt3a was remarkably decreased when compared with the AAV-CaMKII-GFP-injected mouse group (GFP, 100.0 ± 14.7%, *n* = 4, Cre, 40.1 ± 14.9%, *n* = 3, *p* = 0.03, unpaired *t* test, Figure 4B). Behaviorally, we found that, three weeks after PBN injection of AAV-Cre or AAV-GFP in naïve *Dnmt3a*^fl/f^ mice, knockdown of PBN Dnmt3a failed to induce any significant changes in nociceptive response (GFP, 1.23 ± 0.16 g, *n* = 6, Cre, 1.66 ± 0.17 g, *n* = 5, *p* = 0.12, unpaired *t* test, Figure 4C); however, it produced significant anxiety-like behavior as measured in OFT (Central time: GFP, 119.0 ± 17.5 s, *n* = 9, Cre, 68.3 ± 10.4 s, *n* = 8, *p* = 0.03; central distance: GFP, 9.22 ± 1.39 m, *n* = 9, Cre, 5.25 ± 0.67 m, *n* = 8, *p* = 0.02, unpaired *t* test, Figure 4D–F). Again, total distance traveled was not affected by the Dnmt3a knockdown (GFP, 63.15 ± 3.57 m, *n* = 9, Cre, 60.16 ± 3.99 m, *n* = 8, *p* = 0.58, unpaired *t* test, Figure 4G). These results suggest that decreased expression level of Dnmt3a in PBN is likely sufficient to cause anxiety-like behavior and further confirm an important role of PBN Dnmt3a in chronic-pain-induced anxiety behavior.

### 3.4. Dnmt3a Modulates Expression of GluA1 in PBN

**Figure 5:**
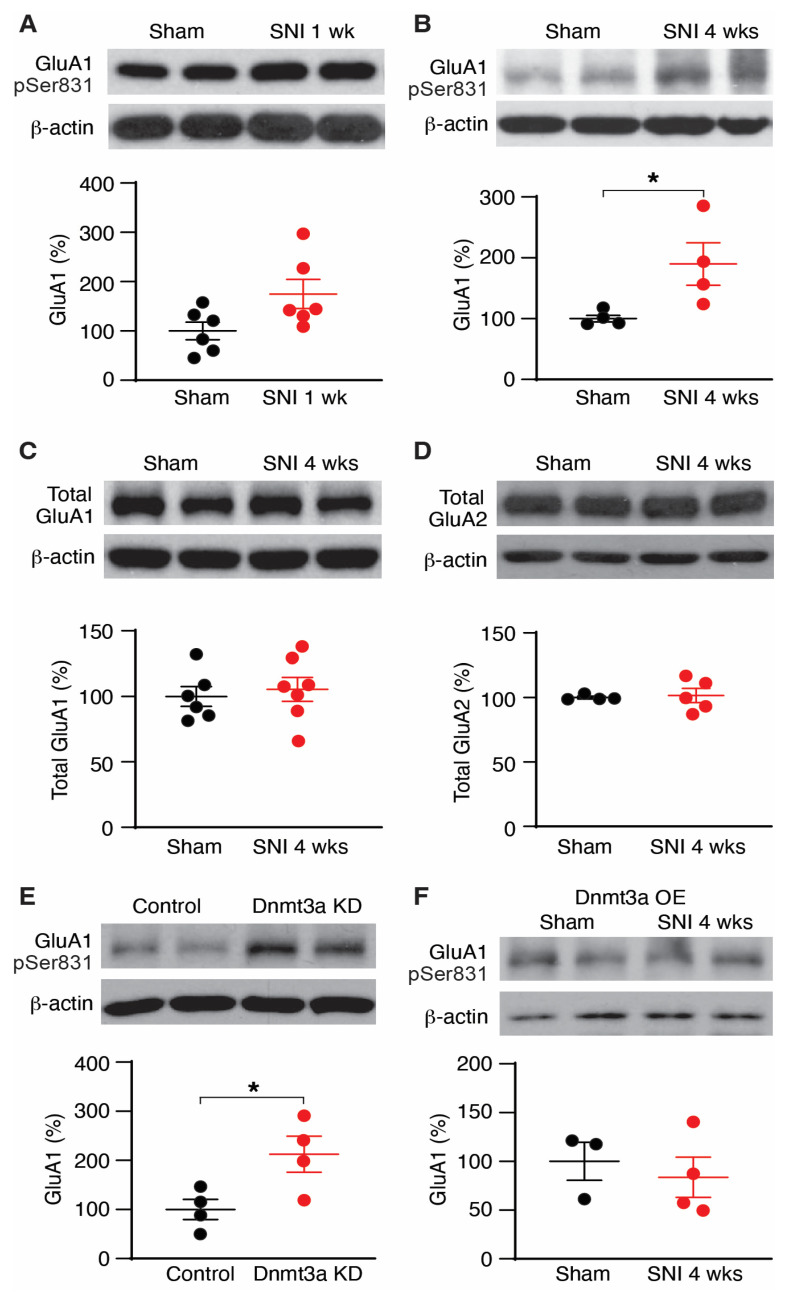
Dnmt3a regulates GluA1 subunits of AMPA receptors in PBN. (**A**–**D**) Western blots and group data of protein levels of GluA1-pSer831 (**A**,**B**), total GluA1 (**C**), and total GluA2 (**D**) normalized to that of b-actin in the PBN from sham-operated and SNI-operated mice 1 week and 4 weeks after the surgery. (**E**) Western blots and group data of normalized GluA1-pSer831 protein levels in the *Dnmt3a*^fl/fl^ mice of AAV-GFP-injected control group and of AAV-Cre-injected Dnmt3a knockdown group 3 weeks after the PBN injections. (**F**) Western blots and group data of normalized protein levels of GluA1-pSer831 in PBN in sham-operated control mice and SNI-operated chronic pain mice (4 weeks after the surgery) with overexpression (OE) of PBN Dnmt3a in both groups (2 weeks after PBN injection of AAV-Dnmt3a). * *p* < 0.05.

Next, we determined potential targets of Dnmt3a modulation in the context of chronic pain. Both peripheral afferents to PBN and PBN projections to other brain areas are primarily glutamatergic [42,43], suggesting a predominant role of glutamate receptors in controlling synaptic activities of PBN neurons and their output effects. Therefore, we examined the PBN expression of GluA1, the dominant subunit of glutamate AMPA receptors crucial for emotional events of learning and memory through activity-dependent synaptic strengthening [44,45,46]. First, we determined whether GluA1 expression was altered in the PBN of mice with chronic pain, focusing on GluA1-pSer831, the GluA1 subunit with phosphorylation at serine 831 that is actively involved in synaptic plasticity and learning [47]. Interestingly, we found that the expression level of GluA1-pSer831 in PBN was significantly increased in the SNI mouse group when compared with the sham group, as follows: at 1 week after the surgery, the increase was obvious and at the borderline of statistical significance (Sham, 100.0 ± 17.9%, *n* = 6, SNI, 174.9 ± 29.4%, *n* = 6, *p* = 0.05, unpaired *t* test, Figure 5A); however, after 4 weeks, the increase became statistically significant (Sham, 100.0 ± 5.3%, *n* = 4, SNI, 189.8 ± 34.9%, *n* = 4, *p* = 0.04, Figure 5B). In contrast, the levels of total GluA1 protein and GluA2 protein were unchanged between the two mouse groups (GluA1: sham, 100 ± 7.6%, *n* = 6, SNI, 105.4 ± 9.1%, *n* = 7, *p* = 0.66; GluA2: sham, 100 ± 1.0%, *n* = 4, SNI, 101.5 ± 5.5%, *n* = 5, *p* = 0.81, unpaired *t* test, Figure 5C,D). Therefore, it is likely that chronic pain increases expression level of the active form of GluA1-pSer831 proteins in PBN, and as a result may increase synaptic function of glutamate AMPA receptors in PBN.

As Dnmt3a-mediated DNA methylation represses gene expression of its targets, we were wondering whether GluA1 was the target of Dnmt3a modulation in PBN and if chronic-pain-induced decrease in Dnmt3a protein level (decreased DNA methylation) mediated the upregulation of GluA1-pSer831 in PBN. First, we mimicked the effect of chronic pain by knocking down the expression of Dnmt3a in PBN and determined its effect on the expression of PBN GluA1-pSer831 in the Cre-dependent *Dnmt3a*^fl/f^ mice. Three weeks after injection of AAV-Cre or AAV-GFP into the PBN of naïve *Dnmt3a*^fl/f^ mice, we found that, after the viral knockdown of PBN Dnmt3a (Figure 4B) that induced anxiety behavior (Figure 4D–F), the PBN level of GluA1-pSer831 proteins was strongly increased (GFP, 100.0 ± 20.5%, *n* = 4, Cre, 212.6 ± 36.5%, *n* = 4, *p* = 0.03, unpaired *t* test, Figure 5E). This indicates that Dnmt3a may change, directly or indirectly, the expression of GluA1-pSer831 and decreased Dnmt3a function could cause the upregulation of GluA1-pSer831 in PBN.

We then determined PBN GluA1-pSer831 level in chronic-pain mice that were rescued from chronic-pain-induced anxiety-like behavior by overexpression of PBN Dnmt3a (Figure 3E–G). We found that the PBN level of GluA1-pSer831 was no longer increased by chronic SNI in these mice without apparent anxiety-like behavior (Sham, 100.0 ± 19.4%, *n* = 3, SNI, 83.6 ± 20.6%, *n* = 4, *p* = 0.6, unpaired *t* test, Figure 5F). This suggests that PBN GluA1-pSer831 is likely involved in the Dnmt3a prevention of chronic-pain mice from developing anxiety behavior.

### 3.5. Knockdown of Dnmt3a Enhances AMPA Synaptic Function

**Figure 6:**
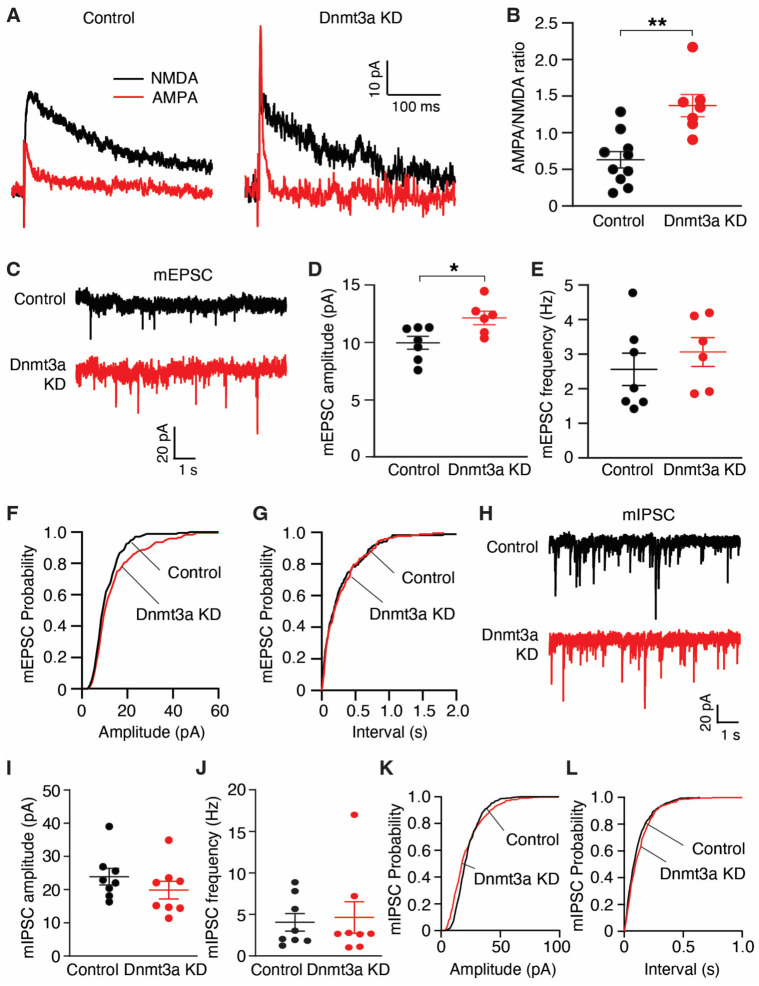
Knockdown of Dnmt3a enhances AMPA synaptic function in PBN. (**A**,**B**) Representative current traces of the AMPA receptor-mediated and the NMDA receptor-mediated excitatory postsynaptic current (EPSC) in a PBN neuron under whole-cell recordings (**A**) and group data of the ratio of AMPA EPSCs over NMDA EPSCs (**B**) from the *Dnmt3a*^fl/fl^ mice of AAV-GFP-injected control group and of AAV-Cre-injected group with knockdown of PBN Dnmt3a 3 weeks after the PBN injections. (**C**–**E**) Current traces (**C**) and group data of the amplitude (**D**) and frequency (**E**) of AMPA miniature EPSCs (mEPSCs) in the *Dnmt3a*^fl/fl^ mice of AAV-GFP-injected control group and of AAV-Cre-injected Dnmt3a knockdown group 3 weeks after the PBN injections. (**F**,**G**) Representative plots of cumulative distributions of AMPA mEPSC amplitude (**F**) and frequency (**G**) from a PBN neuron in the *Dnmt3a*^fl/fl^ mice of above-mentioned control group and PBN Dnmt3a knockdown group. (**H**–**L**) Current traces (**H**), group data of the amplitude (**I**) and frequency (**J**), and representative cumulative distributions of amplitude (**K**) and frequency (**L**) of the GABA_A_ receptor-mediated miniature inhibitory postsynaptic current (mIPSC) in the *Dnmt3a*^fl/fl^ mice of the control group and of the Dnmt3a knockdown group 3 weeks after the PBN injections. * *p* < 0.05, ** *p* < 0.01.

To verify the physiological effect of the increased GluA1-pSer831 at the cellular level, we obtained brain slices containing PBN from naïve *Dnmt3a*^fl/f^ mice 3 weeks after PBN injection of AAV-Cre or AAV-GFP in these mice and performed whole-cell recordings of the AMPA receptor-mediated excitatory postsynaptic current (EPSC) in PBN neurons in vitro to determine changes in AMPA synaptic function after knockdown of PBN Dnmt3a. We first determined changes in the amplitude of evoked AMPA EPSCs in relation to that of NMDA receptor-mediated EPSCs. We found that the ratio of AMPA/NMDA EPSCs was significantly increased in PBN neurons from mice with the Dnmt3a knockdown (GFP, 0.63 ± 0.11, *n* = 10, Cre, 1.37 ± 0.15, *n* = 7, *p* < 0.01, unpaired *t* test, Figure 6A,B), suggesting that knockdown of Dnmt3a augments the function of postsynaptic AMPA receptors in PBN neurons. We then examined action potential-independent AMPA miniature EPSCs (mEPSCs) and found that PBN neurons from the mice with Dnmt3a knockdown displayed significantly larger amplitude of AMPA mEPSC than those from control mice; in contrast, the frequency of AMPA mEPSC was not affected by the Dnmt3a knockdown (Amplitude: GFP, 9.97 ± 0.56 pA, *n* = 7, Cre, 12.15 ± 0.57 pA, *n* = 6, *p* = 0.01; frequency: GFP, 2.54 ± 0.46 Hz, *n* = 7, Cre, 3.04 ± 0.41 Hz, *n* = 6, *p* = 0.19, Kolmogorov–Smirnov test, Figure 6C–G). The increase in mEPSC amplitude but not in mEPSC frequency after Dnmt3a knockdown suggests that the effect is mainly due to enhanced function of postsynaptic glutamate receptors [48]. These results further confirm that Dnmt3a knockdown likely results in upregulated synaptic function of postsynaptic AMPA receptors in PBN.

We also determined whether Dnmt3a regulated the expression of inhibitory GABA_A_ receptors that normally control the activity of excitatory synapses. We found no significant differences in the amplitude or frequency of the GABA_A_ receptor-mediated miniature inhibitory postsynaptic current (mIPSC) between the control and Dnmt3a knockdown groups (Amplitude: GFP, 23.90 ± 2.49 pA, *n* = 8, Cre, 19.85 ± 2.66 pA, *n* = 8, *p* = 0.28; frequency: GFP, 4.04 ± 1.05 Hz, *n* = 8, Cre, 4.63 ± 1.89 Hz, *n* = 8, *p* = 0.79, Kolmogorov–Smirnov test, Figure 6H–L). This indicates that the GABAergic inhibitory synaptic function is not affected by the Dnmt3a knockdown in PBN.

### 3.6. PBN GluA1 Mediates Anxiety-like Behavior

**Figure 7:**
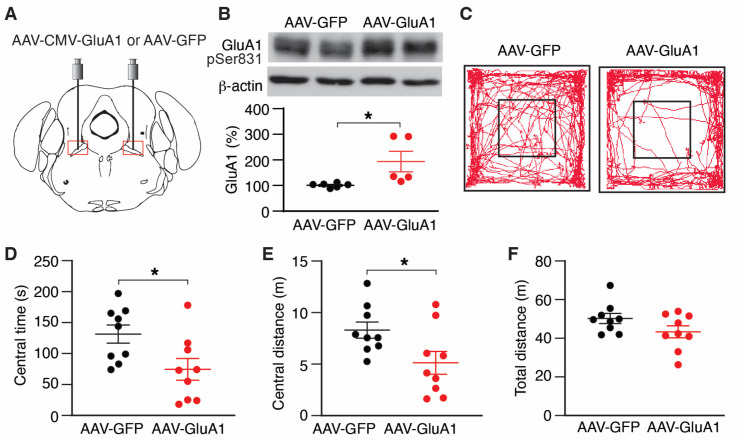
Upregulation of PBN GluA1 induces anxiety-like behavior. (**A**) A diagram illustrating bilateral injection of the viral vector AAV-CMV-GluA1 or the control vector AAV-CMV-GFP into the PBN of naive mice. (**B**) Western blots and group data of normalized GluA1-pSer831 protein levels in mice with PBN injection of AAV-GFP for control or AAV-GluA1 for overexpression of PBN GluA1 3 weeks after the PBN injections. (**C**–**F**) Locomotion traces of a mouse (**C**), and group data of time spent (**D**) and distance traveled (**E**) in central zone, and total distance traveled in the entire arena (**F**) in OFT from naïve mice of the AAV-GFP-injected control group and the AAV-GluA1-injected GluA1 overexpression group 3 weeks after the PBN injections. * *p* < 0.05.

Next, we determined whether GluA1 activity in PBN could lead to anxiety-like behavior by manipulating the expression level of GluA1 in PBN. We first used a viral vector to overexpress local GluA1 in PBN and examined its behavioral effect on anxiety. We found that, 3 weeks after bilateral injection of AAV-CMV-GluA1 into the PBN of naïve mice (Figure 7A), the expression level of GluA1-pSer831 was significantly increased when compared with the AAV-CMV-GFP-injected control group (AAV-GFP, 100.0 ± 3.4%, *n* = 6, AAV-GluA1, 193.6 ± 40.1%, *n* = 5, *p* = 0.03, unpaired *t* test, Figure 7B). It is of note that overexpressing GluA1 protein cannot directly increase GluA1 phosphorylation and might increase the GluA1-pSer831 level likely by increasing the GluA1 pool of phosphorylation substrates. In OFT, the AAV-CMV-GluA1-injected mice displayed significant anxiety-like behavior with decreased central time and central distance, but no change in total distance traveled (Central time: AAV-GFP, 131.4 ± 14.6 s, *n* = 9; AAV-GluA1, 74.4 ± 17.5 s, *n* = 9, *p* = 0.02; central distance: AAV-GFP, 8.30 ± 0.78 m, *n* = 9; AAV-GluA1, 5.12 ± 1.10 m, *n* = 9, *p* = 0.03; total distance: AAV-GFP, 50.24 ± 2.60 m, *n* = 9; AAV-GluA1, 43.31 ± 3.10 m, *n* = 9, *p* = 0.10, unpaired *t* test, Figure 7C–F). Thus, it seems that upregulated expression of GluA1-pSer831 in PBN is likely sufficient to cause anxiety behavior in mice.

**Figure 8:**
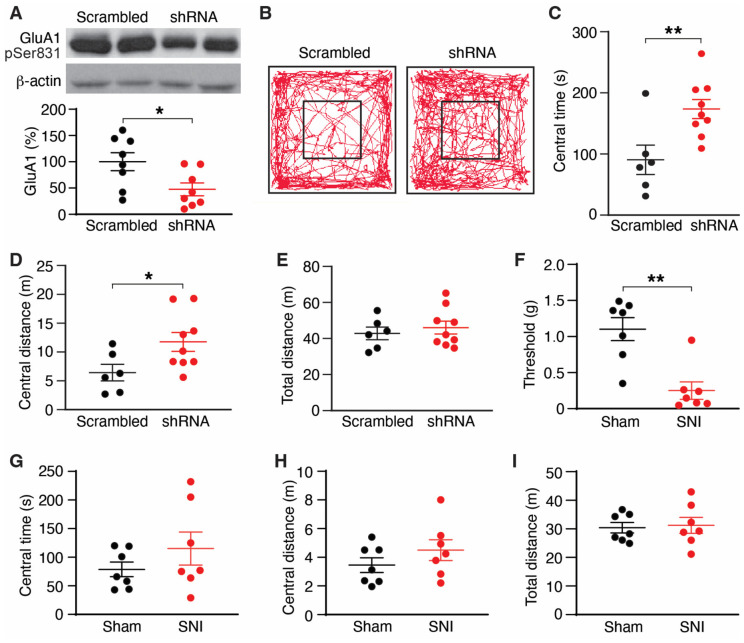
Knockdown of PBN GluA1 blocks pain-induced anxiety-like behavior. (**A**) Western blots and group data of normalized protein levels of PBN GluA1-pSer831 in mice of control group with PBN injection of scrambled GluA1-shRNA and of GluA1 knockdown group with PBN injection of GluA1-shRNA 3 weeks after the PBN injections. (**B**–**E**) Locomotion traces of a mouse (**B**), and group data of time spent (**C**) and distance traveled (**D**) in central zone, and total distance traveled in the entire arena (**E**) in OFT in mice of the scrambled GluA1-shRNA-injected control group and GluA1-shRNA-injected GluA1 knockdown group 3 weeks after the PBN injections. (**F**) Pain thresholds in sham and SNI mice 3 weeks after bilateral injection of GluA1-shRNA into PBN. (**G**–**I**) Group data of time spent (**G**) and distance traveled (**H**) in central zone, and total distance traveled in the entire arena (**I**) in OFT in sham and SNI mice 3 weeks after bilateral injection of GluA1-shRNA into PBN. * *p* < 0.05, ** *p* < 0.01.

We then used an shRNA vector to knock down GluA1 in PBN and examined its behavioral effect in naïve mice. Three weeks after bilateral injection of AAV-GluA1-shRNA into the PBN, our Western blot results showed significantly decreased expression of GluA1-pSer831 in PBN when compared with the control group injected with AAV-GluA1-scrambled shRNA (Scrambled, 100.0 ± 17.1%, *n* = 8, shRNA, 47.6 ± 12.1%, *n* = 8, *p* = 0.02, unpaired *t* test, Figure 8A). Behaviorally in OFT, we found that the GluA1 knockdown group showed a significant decrease both in central time and in central distance with total distance unchanged when compared with the control group, indicating that knockdown of GluA1-pSer831 in PBN can induce an anxiolytic effect in normal conditions (Central time: scrambled, 90.5 ± 24.0 s, *n* = 6, shRNA, 173.5 ± 15.6 s, *n* = 9, *p* < 0.01; central distance: scrambled, 6.43 ± 1.43 m, *n* = 6, shRNA, 11.76 ± 1.64 m, *n* = 9, *p* = 0.03; total distance: scrambled, 42.83 ± 3.52 m, *n* = 6, shRNA, 46.04 ± 3.56 m, *n* = 9, *p* = 0.55, unpaired *t* test, Figure 8B–E).

Finally, we examined whether PBN GluA1 was involved in chronic-pain-induced anxiety behavior. PBN GluA1 proteins were knocked down as above by bilateral injection of AAV-GluA1-shRNA into the PBN in both sham and SNI mice. We found that, after the knockdown of PBN GluA1, SNI still induced significant nociceptive hypersensitivity 4 weeks after the surgery (Threshold: sham, 1.10 ± 0.15 g, *n* = 7, SNI, 0.25 ± 0.12 g, *n* = 7, *p* < 0.01, Figure 8F); however, the SNI failed to induce significant anxiety-like behavior, with no change in locomotor activity in SNI mice when compared with sham mice (central time: sham, 78.7 ± 12.8 s, *n* = 7, SNI, 115.2 ± 28.8 s, *n* = 7, *p* = 0.26; central distance: sham, 3.45 ± 0.51 m, *n* = 7, SNI, 4.50 ± 0.72 m, *n* = 7, *p* = 0.25; total distance: sham, 30.38 ± 1.83 m, *n* = 7, SNI, 31.24 ± 2.79 m, *n* = 7, *p* = 0.79, Figure 8G-I). These results suggest that GluA1 of AMPA receptors in PBN may be required in chronic-pain-induced anxiety behavior.

## 4. Discussion

In this study, we have provided molecular, cellular, and behavioral evidence showing that Dnmt3a in PBN is an important regulatory protein in mediating chronic-nerve- injury-induced behaviors of negative emotion in mice. Thus, local overexpression of PBN Dnmt3a can overcome the Dnmt3a down-regulation induced by nerve injury and rescue the mice from developing anxiety-like behavior, while knockdown of PBN Dnmt3a appears sufficient to cause anxiety behavior in naïve conditions, mimicking the effect of nerve injury. Furthermore, we have shown that the GluA1 subunit of glutamate AMPA receptors could be a potential target of Dnmt3a in PBN in chronic-pain-induced anxiety, as knockdown of Dnmt3a increases GluA1 protein level and GluA1 synaptic function in PBN, altering PBN GluA1 levels changes the level of anxiety behavior in a positive relationship, overexpression of Dnmt3a overwhelms chronic-pain-induced GluA1 increase in PBN with subdued anxiety behavior, and knockdown of PBN GluA1 prevents chronic- pain-induced anxiety behavior. These results support an epigenetic mechanism for chronic-pain-anxiety comorbidity so that chronic pain downregulates Dnmt3a, which upregulates GluA1 expression and enhances synaptic function of AMPA receptors in PBN, leading to anxiety behavior through activated PBN projections.

Epigenetic regulations of gene expression have drawn increasing interest in recent studies of neurobiological mechanisms of pain in different animal models [20,39,49,50,51,52,53]. Dnmt3a-mediated DNA methylation for gene repression has been implicated in the mechanisms for sensitized conditions of sensory pain. For example, in peripheral afferent neurons and spinal cord neurons, Dnmt3a is involved in the epigenetic mechanism underlying nerve-injury-induced sensitization of neuropathic and inflammatory pain [29,32,54,55,56]. In the brain, recent studies have shown that Dnmt3a is downregulated or global methylation level is decreased in amygdala and the prefrontal cortex (PFC) in a mouse model of chronic neuropathic pain (after 6 months of pain presence) [57,58], but another study shows no change in the Dnmt3a level in amygdala, PFC, and ventral hippocampus in the presence of short-term persistent inflammatory pain (10 days of pain presence) in rats [59]. It is likely that Dnmt3a levels display dynamic changes during the period of chronic pain development, but the timeline of Dnmt3a activity after pain induction is unclear and has not been systemically studied. These dynamic Dnmt3a changes are likely time- and region-dependent in the central nervous system. It appears that long-term, chronic pain conditions may be required to induce significant changes in Dnmt3a levels in the brain. The current study provides original evidence that directly links Dnmt3a to chronic-pain-induced emotional effects, suggesting a DNA methylation-related epigenetic mechanism in PBN for the common comorbidity of chronic pain and negative emotion.

The role of Dnmt3a in emotional disorders has been reported in animal models of another aversive condition–stress. Chronic social defeat stress (CSDS) increases Dnmt3a expression in nucleus accumbens (NAc), and Dnmt3a overexpression in NAc induces depression-like behavior in mice [60]. However, in medial PFC, CSDS decreases Dnmt3a expression and Dnmt3a knockdown induces anxiety-like behavior in mice [61]. In contrast to the current findings of chronic-nerve-injury-induced Dnmt3a decrease in mice, chronic traumatic brain injury that induces anxiety-like behavior in rats increases Dnmt3a expression in amygdala [62]. Thus, while Dnmt3a is important for the regulation of emotion-related behaviors, its functions and mechanisms appear to be region-specific in the brain.

Despite numerous studies on roles of Dnmt3a and DNA methylation in general in chronic neurologic diseases including chronic pain, the gene targets of DNA methylation in the pathogenesis of these diseases remain largely unclear. It has been shown that Dnmt3a likely regulates the expression of brain-derived neurotrophic factor (BDNF) gene in amygdala in rat models of stress [62,63]. Dnmt1 has been reported to downregulate glutamic acid decarboxylase 67 (GAD67) of GABAergic neurons in the CeA of rats with pain-induced depression-like behavior and in the basolateral amygdala of prenatally stressed mice [64,65]. These studies generally suggest an upregulation of Dnmts and resulted hypermethylation of BDNF and GAD67 genes in inhibitory GABAergic neurons in animal models of stress and pain. The current study has shown that, in PBN, of which over 90% of neurons are excitatory glutamatergic neurons [43], Dnmt3a may regulate, directly or indirectly, the expression of GluA1 subunits of glutamate AMPA receptors in the process of chronic-pain-induced anxiety behavior. Although the detailed methylation mechanisms for Dnmt3a regulation of glutamate GluA1 subunits remain to be investigated, our findings provide original evidence for the possibility of the excitatory GluA1 subunit as a regulation target of Dnmt3a in the brain. While this study focused on GluA1 subunits of AMPA receptors, NR2B-containing NMDA receptors are also involved in synaptic plasticity in the brain and can affect phosphorylation and trafficking of GluA1-containing AMPA receptors [44,46]. The contribution of NR2B-containing NMDA receptors to activity of PBN neurons in chronic pain would be an interesting topic for future studies.

Chronic pain is often associated with comorbid behaviors of negative emotion including anxiety and depression in both patients and in animals [66,67]. For PBN relay of affective pain of negative emotion, glutamatergic neurons in PBN project to several brain areas and the excitatory projections have been shown to mainly promote behaviors of negative affective states [68,69]. Specifically, activation of lateral PBN projections to the ventromedial hypothalamus, the bed nucleus stria terminalis, or CeA generates an aversive memory or escape behavior, but only activation of dorsal PBN projections to the lateral periaqueductal gray inhibits nociceptive pain [70]. The PBN to CeA projection has been shown to carry the affective aspect of pain signal to create threat memory, and optogenetic activation of this projection induces behaviors of negative emotion [17,18]. Based on our data, we hypothesize that chronic pain downregulates Dnmt3a, leading to upregulation of active GluA1 through increased phosphorylation and enhanced synaptic activity of AMPA receptors in PBN; increased AMPA receptor activity activates PBN glutamatergic neurons and their projections to CeA (and other forebrain regions), ultimately causing aversive behaviors including anxiety and depression. This represents our current working model and could be one of many interacting pathways involved in the Dnmt3a function in chronic-pain-associated aversive behaviors. How local circuits in CeA (and other PBN projection regions) and downstream steps beyond these PBN targets mediate the aversive behaviors remains largely unclear. These would be highly intriguing topics for future studies in the brain.

It is interesting to note our current results that knockdown of PBN Dnmt3a that was sufficient to induce anxiety-like behavior did not affect baseline pain response in naïve mice, and overexpression of PBN Dnmt3a rescued the mice from pain-induced anxiety behavior but not from the nociceptive hypersensitivity itself. This is consistent with previous reports that activating the PBN–CeA pathway does not change nociceptive responses [18,70,71]. However, a recent study showed that, while the PBN–CeA pathway did not alter baseline nociception, it played a critical role in short-term nerve-injury-induced hypersensitivity of nociception [14]. It is possible that the longer period (>4 weeks) and the degree of nerve injury in the present study might be among the reasons for the lack of effect of PBN Dnmt3a overexpression on the SNI-induced nociceptive hypersensitivity we observed. Overall, it seems that activation of the excitatory PBN projections in the brain predominantly mediates negative affective behaviors rather than baseline pain responses although it may also induce acute stress- or acute threat-induced analgesia [72,73].

In summary, we have shown that Dnmt3a in PBN functions as a key epigenetic modulator in the mechanisms of chronic-pain-induced behaviors of negative emotion, and GluA1 subunits of AMPA receptors may be a potential target of the Dnmt3a modulation in PBN. As overexpression of PBN Dnmt3a and knockdown of PBN GluA1 proteins can rescue mice from developing anxiety behavior, it raises the possibility that Dnmt3a may serve as a molecular target for the development of future therapeutic strategies to improve the currently challenging treatment of chronic pain and comorbid emotion disorders.

## Data Availability

The original data of this study are available from the corresponding author upon request.
